# The adaptation of the GameSquad exergaming intervention for young adults with Down syndrome: A pilot feasibility study

**DOI:** 10.1016/j.dhjo.2024.101766

**Published:** 2024-12-06

**Authors:** Kameron Suire, Brian C. Helsel, April Bowling, Amanda E. Staiano, Joseph R. Sherman, Anna Rice, Lauren T. Ptomey

**Affiliations:** a Department of Kinesiology, Berry College, 2277 Martha Berry Hwy NW Mount Berry, GA, 30149, USA; b University of Kansas Alzheimer’s Disease Research Center, Department of Neurology, University of Kansas Medical Center, 3901 Rainbow Blvd, Kansas City, KS, 66160, USA; c School of Nursing and Health Sciences, Merrimack College, 315 Turnpike Street North Andover, MA, 01845, USA; d Pennington Biomedical Research Center, Louisiana State University, 6400 Perkins Rd, Baton Rouge, LA, 70808, USA; e Division of Physical Activity and Weight Management, Department of Internal Medicine, University of Kansas Medical Center, 3901 Rainbow Blvd, Kansas City, KS, 66160, USA

**Keywords:** Exercise, Intellectual disabilities, Clinical trial, Energy expenditure

## Abstract

**Background:**

Exergames may be a feasible alternative to in-person exercise that is adaptable for adults with Down Syndrome (DS).

**Objective:**

The purpose of this study was to conduct a 12-week pilot trial to assess the feasibility of exergames for adults with DS.

**Methods:**

Adults with DS were provided Ring Fit Adventure^™^ which uses a resistance ring and body weight to perform cardiovascular and strength exercises. Participants were instructed to play Ring Fit Adventure^™^ for 120 min/week and attend 30-min weekly virtual coaching sessions where a health educator encouraged gameplay via goal setting, helped troubleshoot technological issues, and collected self-reported minutes of gameplay. Intervention outcomes included attendance, adherence to weekly gameplay goals, retention, safety, and exercise intensity captured via heart rate and indirect calorimetry.

**Results:**

Twenty adults with DS (age 23.5 years, 89 % non-Hispanic white, 61 % female) enrolled and 19 participants completed the trial. Participants attended 93 % of coaching sessions and 90 % met the weekly gameplay goals. The average gameplay duration was 39 min/session and 123 min/week at 67.3 % of the participants’ estimated maximum heart rate. Both the average heart rate during the intervention and metabolic equivalents (3.4 ± 1.0) during the indirect calorimetry assessment were suggestive of moderate intensity exercise.

**Conclusions:**

Attendance and adherence to the weekly gameplay goal were high among adults with DS who were able to reach and sustain moderate intensity during the exergame sessions. Exergaming represents a home-based option for accumulating minutes of moderate-to-vigorous physical activity that is feasible for and acceptable to adults with DS.

## 1. Introduction

Down Syndrome (DS) or trisomy 21 is the most common chromosomal abnormality associated with intellectual disabilities (ID).^1^ The life expectancy for people with DS has increased dramatically over the past 60 years due to advancements in disease knowledge, medical care, and caregiving services,^2^ creating a critical need to address ways to support healthy living throughout the lifespan. Increased moderate-to-vigorous physical activity (MVPA) is associated with improved health outcomes and physical function among individuals with DS.^3^ However, it is estimated that only 9 % of adults with ID, including those with DS,^4^ achieve 150 min/week of MVPA as recommended by the Physical Activity Guidelines for Americans.^5^ Population-based studies from the general population, and limited evidence from research in individuals with DS, suggest that the 7-year transitional period between adolescence and adulthood^6-13^ is associated with decreased physical activity^9^ and may be a critical period for intervention.

The few trials evaluating strategies for increasing MVPA in young adults with ID have been unsuccessful due to the interventions being short-term (i.e., insufficient for behavior change and developing habits) and lacking the activity intensity needed to accumulate minutes of MVPA.^6,14,15^ Additionally, young adults with DS face unique challenges to increasing MVPA including gait abnormalities^7^; low self-confidence, social support, and self-efficacy; and a lack of affordable/accessible transportation to facilities focused on creating adaptable MVPA opportunities.^8^ Exergames, which integrate physical activity into video game play, represent an affordable, accessible, and non-traditional home-based exercise modality that has been shown to elicit MVPA^13,16,17^ and improve physical function in a variety of populations.^18-20^ Exergaming holds promise for adults with DS by enhancing intrinsic motivation, promoting self-determination, and fostering autonomy without needing a deep understanding of physical activity benefits.^21^ It provides ample opportunities for repetition and real-time feedback,^22^ while also aligning with the visuospatial learning preferences of adults with DS—the ability to create, recall, and interpret visual images.^23,24^ Additionally, exergaming delivers an immersive storytelling experience with clear goals and diverse activities to maintain engagement and prevent monotony.

GameSquad is an exergaming intervention originally developed and tested in 46 adolescents with overweight/obesity resulting in reduced body mass index (BMI) z-scores, improvements in cardiometabolic biomarkers, and increased MVPA.^25^ A subsequent adaptation of GameSquad was successfully completed in adolescents with neurodevelopmental and psychiatric diagnoses.^26^ The purpose of this study was to adapt GameSquad for adults with DS (GameSquad-DS) using the exergaming platform Ring Fit Adventure^™^ and determine the initial feasibility of GameSquad-DS across a 12-week pilot trial. We aimed to assess the retention, attendance, adherence, safety, and intensity of the exergaming sessions during the GameSquad-DS intervention.

## 2. Methods

### 2.1. Overview

This single-arm study was approved by the University Institutional Review Board for Research Involving Human Subjects and is registered on clinicaltrials.gov (NCT05473247). The intervention was conducted from January to June 2023 in the United States.

### 2.2. Participants

The inclusion criteria for the study were as follows: 1) Young adults (18–30 yrs); 2) Diagnosis of DS as determined by a Community Service Provider operating in our recruitment area under the auspices of a Community Developmental Disability Organization; 3) Sufficient functional ability to understand directions, communicate preferences, wants, and needs through spoken language; and 4) Living at home with a parent/guardian or in a supported living environment with a parent/caregiver who agrees to serve as a study partner. Participants were excluded if they self-reported or had parent reported: 1) inability to participate in MVPA or 2) a serious medical risk, e.g., cancer, recent heart attack, stroke, angioplasty as determined by their primary care provider.

### 2.3. Intervention

The original GameSquad used the Xbox Kinect, which was discontinued in 2017. Thus, for GameSquad-DS, we selected a different game called Ring Fit Adventure^™^ played on the Nintendo Switch^™^ gaming system (Nintendo Co., Ltd, Kyoto, Japan). Ring Fit Adventure^™^ uses both a resistance ring and body weight to perform upper and lower body cardiovascular and strength exercises. In contrast to other exergames, participants incorporate exercise to complete an adventure narrative guided by Ring Fit Adventure^™^ characters. The narratives provide ~60 h of unique activity which increases in both intensity and difficulty as participants progress through the game. Participant exercise is guided by both written instructions and visual demonstrations via an in-game avatar. Feedback regarding proper exercise form is provided by controllers in the game ring containing imbedded accelerometers to monitor movement. The game difficulty was initially set to 3 out of 30, with a lower difficulty reducing the number of exercise repetitions required to complete the tasks but not altering the fundamental movements used during gameplay.

Young adults with DS were asked to play Ring Fit Adventure^™^ for a minimum of 120 min/week over 12 weeks and attend 12 weekly, 30-min virtual health coaching sessions on Zoom^®^ alongside their parent/caregiver. During the health coaching sessions, trained health educators reviewed participation data from the most recent week, presented information on the importance of engaging in MVPA, conducted troubleshooting for technical issues, and provided social support and encouragement. Gameplay goals increased progressively during the first 4 weeks of the intervention starting at a minimum of 60 min during week 1 and escalating to 80 min during week 2, 100 min during week 3, and 120 min by week 4. Participants were taught how to change the gameplay difficulty and enable various built-in accessibility options, known as “assist modes”, designed to accommodate individuals with disabilities.

Participants started with the first level of the game and progressed forward through numerous levels and worlds at their own pace. Each level required approximately 10–15 min to complete, and each world has about 5–10 different levels. Participants moved through the level by moving their body (running, rowing, flying, etc.) and battling against in-game “monsters” using various exercises that involved the Pilates style ring and leg-strap (squats, overhead ring-press, bow-pulls, etc.). Participants unlocked new exercises and characters as they completed levels and defeated monsters to reward progression.

### 2.4. Outcomes

Attendance was assessed as the percentage of the 12 weekly 30-min health coaching sessions attended as recorded in the health coach reports.

Intervention adherence was assessed by calculating the percentage of the 12 weekly gameplay goals completed as prescribed. This was based on both the number of minutes per week of gameplay recorded by the Fitbit and self-reported gameplay goals.

Retention was measured as the percentage of participants retained throughout the intervention.

Safety was determined by the number and type of serious adverse events.

#### Anthropometrics.

Weight was assessed to the nearest 0.1 kilogram (kg) using a calibrated scale (Model #PS660, Belfour, Saukville, WI). Standing height was measured to the nearest centimeter (cm) using a portable stadiometer (Model #IP0955, Invicta Plastics Limited, Leicester, UK). BMI was calculated as weight in kilograms divided by height in meters squared (kg/m^2^). Weight and height were assessed in duplicate with a third measurement taken if there was a discrepancy ≥0.1 pounds (0.05 kg) or 0.25 inches (0.635 cm).

#### Session intensity.

Participants were given a Fitbit Versa 2^™^ (Fitbit LLC, San Francisco, CA) wireless activity monitor with instructions to capture their workouts when using Ring Fit Adventure^™^. Specifically, they were instructed to select the “Workout” option on the Fitbit Versa 2^™^ to start an exercise session right before the in-game warm-up and to end the workout immediately after the in-game cool-down. This allowed for heart rate (HR) to be recorded within the workout and provided participants an opportunity to self-monitor the time of their workouts to increase adherence to the 120-min weekly gameplay goal. Data from the sessions were extracted from the Fitbit API using an R package developed by our team (https://github.com/bhelsel/iFitbit). Average HR during the session and percent of maximal HR (HR_max_) using a DS-specific equation (210 – 0.56 x age – 31)^27^ were used to measure session intensity.

Energy expenditure of gameplay was assessed using a COSMED K5 (Pensacola, FL) indirect calorimeter. This previously validated,^28-30^ lightweight (~1.5 kg) open-circuit portable indirect calorimeter measures breath-by-breath ventilation and concentration of expired oxygen and carbon dioxide. HR was assessed by a Garmin HR monitor connected via Bluetooth^®^ to the COSMED K5. The energy expenditure sessions took place at the end of the 12-week intervention, within a laboratory under the supervision of staff, and all participants completed the same two levels of gameplay. The two levels of gameplay selected were “Nightcloak Pass” and “Dashalong Tower”, the first two levels of “World 2 - The Land of the Night”. We selected these levels based on participant familiarity (as they occur early in the game) and because they involve multicomponent exercises that target aerobic and muscular systems. These exercises include running; knee lifts; front, abdominal, and overhead presses; squats; and knee-to-chest movements and gameplay difficulty was set at 5 out of 30. The energy expenditure data was downloaded in 30-s epochs and metabolic equivalents (METs) were used to estimate exercise intensity with 3 and 6 METs used to characterize thresholds for moderate and vigorous intensity, respectively.

### 2.5. Statistical analysis

Data are presented as mean ± standard deviation for continuous measures and frequency (percentage) for categorical variables. Intervention adherence is described as the average percentage of coaching sessions attended and gameplay goals reached across the 12-week intervention. Activity and heart rate data collected from the Fitbit Versa 2^™^ is reported for participants who recorded at least 5 gameplay sessions ≥10 min in duration and visualized using a raincloud plot. Boxplots were used to display METs from the energy expenditure assessments. The data processing and visualization was conducted using R (v4.3.2).^31^

## 3. Results

Twenty adults with DS enrolled in the intervention and 19 participants (95 %) completed the study. One participant was lost to follow-up after completing the pre-testing appointment and never started the intervention. Sample characteristics of those enrolled in the study are shown in Table 1. Participants were 23.5 ± 4.0 years of age, 61 % female, 89 % non-Hispanic white, and had a mean BMI of 32.1 ± 7.7 kg/m^2^. Nearly all of the sample had overweight (n = 8; 40 %) or obesity (n = 11; 55 %).

### Intervention Adherence.

Participants attended 93 ± 10 % (11.2 ± 1.2) of the 12 coaching sessions. Across all participants, only 15 coaching sessions were missed, and 7 of these sessions (47 %) were because of illness as reported by the participant or parent. No serious adverse events were reported by the participants (or the parents on behalf of the participants) throughout the duration of the study. On average, participants achieved the self-reported gameplay goals 90 ± 11 % (10.8 ± 1.3 weeks) of the time during the 12-week intervention. The percentage of participants meeting the gameplay goal by week ranged from 58 % to 100 %. Ten (45 %) of the 22 missed weekly gameplay goals across all participants were due to illness.

### Fitbit HR Data.

Sixteen participants recorded 581 exercise sessions ≥10 min across the 12-week intervention (Table 1; 22–57 sessions across 7–12 weeks). Three participants were excluded from this part of the analysis due to the limited amount of Fitbit heart rate data collected (i.e., <5 gameplay sessions). The average HR for adults with DS across these sessions was 111 ± 14 beats per minute which was 67.3 ± 8.2 % of their estimated HR_max_. The average percent of each participant’s HR_max_ across all measured intervention sessions was ≥64 % HR_max_ for 69 % of the participants (n = 11 out of 16) and ≥76 % HRmax for 13 % of the participants (n = 2 out of 16) which are the criteria for achieving moderate and vigorous intensity as defined by the American College of Sports Medicine (ACSM).^32^ Overall, the average percent of HR_max_ during gameplay reached ACSM’s MVPA criteria during 66 % of the recorded sessions. The average duration of the exercise sessions was 39 min (123 ± 33 min/week). Participants spent an average of 16 % (6.3 min) and 26 % (10.3 min) of their time during gameplay accumulating fairly active and very active minutes as categorized by the Fitbit Versa 2^™^. This accumulated to 58 ± 42 min/week of gameplay spent in fairly or very active minutes (range: 7–132 min/week) with 6 out of 16 participants reaching an average of 75 min/week or higher across the 12-week intervention.

**Fig. 1.**
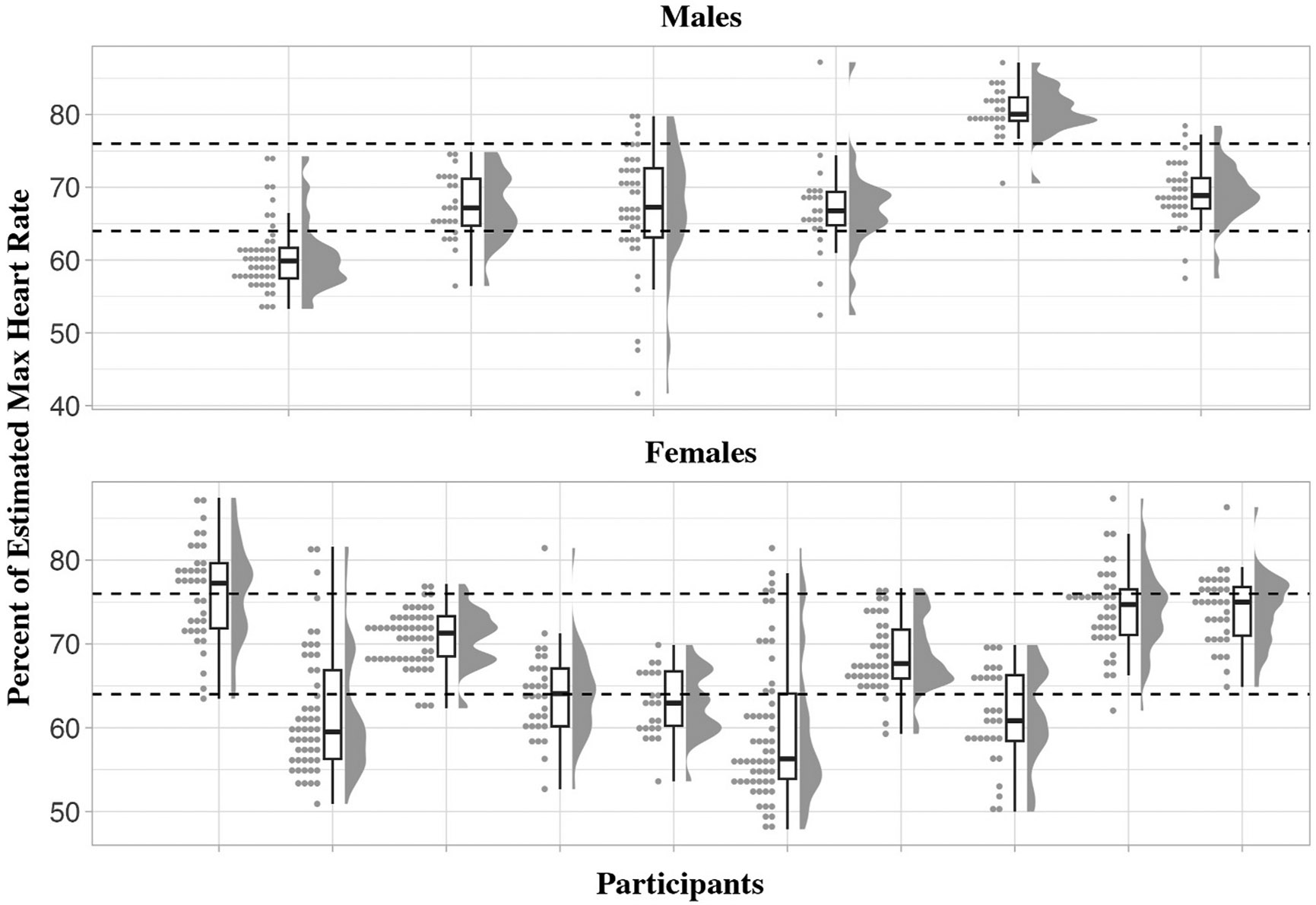
Percent of estimated maximal heart rate measured by the fitbit versa 2^™^ across the 12-week GameSquad-DS intervention.

Raincloud plots displaying the average percent of estimated max heart rate and the distribution across the recorded sessions is displayed in Fig. 1. Overall, median values for 11 of the 16 participants (5 males, 6 females) were consistently at or above moderate intensity as characterized by ACSM. The horizontal dashed lines in Fig. 1 represent the ACSM classification of moderate (≥64 % HR_max_) and vigorous (≥76 % HRmax) intensity.

### Energy Expenditure.

**Fig. 2.**
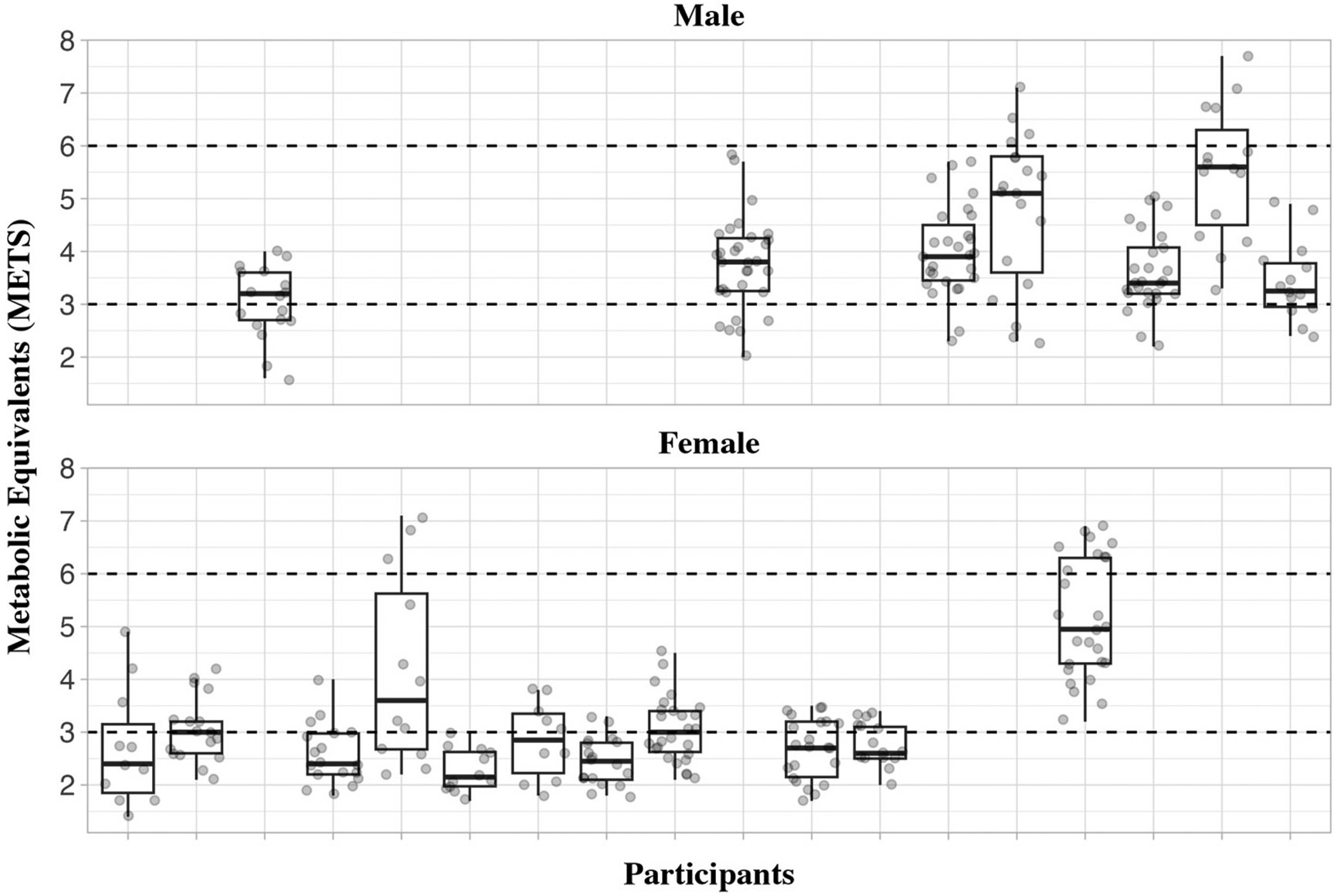
Individual variability in metabolic equivalents assessed by indirect calorimetry over 30-second epochs during two levels of ring fit Adventure^™^ gameplay.

Eighteen participants (95 %) completed the energy expenditure assessment at the end of the 12-week intervention. The energy expenditure assessment lasted ~9 min (range: 4.5–15 min) with participants completing the two levels of gameplay at an average HR of 105 ± 18 beats per minute (Percentage of HR_max_ 63.7 ± 10.7 %). The average METs for the assessment was 3.4 ± 1.0 (VO2: 12.0 ± 3.4 ml/kg/min.) which is consistent with exercising at a moderate intensity. Fig. 2 shows the median METs and the individual variability in METs averaged over 30-s epochs while participants were completing the energy expenditure assessment. The horizontal dashed lines are drawn to represent moderate (3 METs) and vigorous (6 METs) intensity.

## 4. Discussion

Adults with DS have low levels of MVPA and face several barriers that increase the difficulty of engaging in interventions for increasing MVPA. Retention, attendance, adherence, and safety data from this pilot trial suggest that a combination of self-led, home-based exergaming and brief weekly support/education sessions delivered via Zoom^®^ may provide a feasible alternative to traditional, on-site approaches for increasing MVPA in adults with DS. Additionally, HR and energy expenditure data suggest the intensity of the gameplay sessions were at or above moderate intensity.

The results of this study are similar to the first iteration of GameSquad conducted among adolescents with overweight and obesity. In the first GameSquad, 94.4 % of the participants adhered to their weekly exergaming goals compared to 90 % in GameSquad-DS. Additionally, attendance at the coaching sessions was 92.7 % in GameSquad which is similar to the 93 % in GameSquad-DS.^25^ In the later adaptation of GameSquad for adolescents with neurodevelopmental and psychiatric diagnoses, the authors reported an average of ~82 min/week of self-reported exergaming with the adolescents attending 83 % of the scheduled health coaching sessions.^26^ The current trial observed higher levels of Fitbit device-reported gameplay (133 min/wk) and higher attendance, suggesting a successful adaptation of exergaming to young adults with DS and a high potential for an adequately powered study exploring the effect of exergaming on health outcomes in this population. Very few studies conducted among adolescents with intellectual disabilities have focused on increasing MVPA which is the exercise intensity associated with health benefits.^5^ A 2018 systematic review and meta-analysis identified only 5 relevant interventions aiming to increase PA in adolescents with intellectual and developmental disabilities.^15^ MVPA increased by 3.6–9.5 min/day, but the meta-analysis showed a lack of effectiveness in increasing MVPA to a level that would decrease chronic disease risk (*d*: 2.20; 95 % CI −0.57 to 0.97).

The use of exergames to increase MVPA among individuals with DS has been used for therapy in patients with DS with promising results for balance (SMD = 2.72; 95 % CI = 1.68–3.76), functional mobility (SMD = 4.14; 95 % CI = 3.69–4.59), and muscle strength (SMD = 6.40; 95 % CI = 2.68–10.11) in a recent meta-analysis and systematic review.^22^ Additionally, an intervention by Perrot et al.^19^ observed that the average HR of 6 adults with DS was ~15 beats per minute lower than the current GameSquad-DS intervention with no participants engaging in moderate intensity exercise. This differs from GameSquad-DS where the HR and energy expenditure data support the accumulation of MVPA during gameplay sessions.^33^

Limitations of this study include: the small sample size of young adults with DS restricts its generalizability; the 12-week intervention’s short duration hinders the adoption of exercise habits; variability in the sessions recorded with the Fitbit prompts questions about the reliability of device-based measures in this population; and the absence of a comparison group in this non-randomized trial impacts its findings. Additionally, the energy expenditure assessment was conducted in a lab-based setting, so it is difficult to know if it is comparable to at-home exergaming. Future randomized trials are needed to assess the impact of the GameSquad-DS intervention on longitudinal changes in MVPA.

## 5. Conclusions

Results of this pilot trial suggest that the delivery of at-home exergaming with remote coaching sessions to young adults with DS is a feasible approach to promote MVPA participation. Adequately powered trials to further evaluate the impact of long-term (e.g. ≥ 6–18 months) at-home exergaming on health outcomes are warranted. This approach shows promise and potential to be a cost-effective, sustainable, and effective solution for increasing or maintaining MVPA in young adults with DS.

## Figures and Tables

**Table 1:** Sample characteristics and ring-fit Adventure^™^ gameplay sessions across the 12-week GameSquad-DS intervention.

Demographics	Overall	Male	Female
	N = 20^a^	N = 7^a^	N = 13^a^
Age	23.5 ± 3.9	23.1 ± 4.1	23.7 ± 4.0
Ethnicity			
Hispanic or Latino	2 (10 %)	0 (0 %)	2 (15 %)
Not Hispanic or Latino	18 (90 %)	7 (100 %)	11 (85 %)
Race			
Native Hawaiian or Pacific Islander	1 (5.0 %)	1 (14 %)	0 (0 %)
Black or African American	1 (5.0 %)	1 (14 %)	0 (0 %)
White	18 (90 %)	5 (71 %)	13 (100 %)
Weight (kg)	73.4 ± 17.1	74.8 ± 5.5	72.7 ± 21.1
Height (cm)	151.1 ± 9.0	159.6 ± 4.0	146.5 ± 7.4
Body Mass Index	32.3 ± 7.8	29.5 ± 3.1	33.8 ± 9.2
Gameplay	N = 581^a^	N = 191^a^	N = 390^a^
Duration (minutes)	39.0 ± 19.2	42.2 ± 21.5	37.4 ± 17.8
Steps	1051 ± 976	1157 ± 1375	999 ± 699
Heart Rate (HR)	111 ± 14	112 ± 13	111 ± 14
Estimated HR Max	166 ± 2	166 ± 2	166 ± 2
Percent of HR Max	67.3 ± 8.2	67.6 ± 8.2	67.2 ± 8.3
Activity Intensity			
Sedentary	21.0 ± 26.5	28.2 ± 36.1	17.5 ± 19.3
Lightly Active	35.5 ± 28.9	32.1 ± 31.5	37.2 ± 27.4
Fairly Active	16.1 ± 17.8	13.1 ± 16.0	17.5 ± 18.4
Very Active	26.3 ± 31.7	25.4 ± 32.7	26.7 ± 31.2
Average Session HR			
Moderate ≥64 % HR Max	385 (66 %)	129 (68 %)	256 (66 %)
Vigorous ≥76 % HR Max	87 (15 %)	31 (16 %)	56 (14 %)

Sedentary time and physical activity are measured as a percentage of the duration.

American College of Sports Medicine (ACSM): Moderate Intensity ≥64 % HR Max; Vigorous Intensity ≥76 % HR Max.

a Mean ± SD; n (%).

## References

[1] Parker SE, Mai CT, Canfield MA, Updated National Birth Prevalence estimates for selected birth defects in the United States, 2004-2006. Birth Defects Res A Clin Mol Teratol. Dec 2010;88(12):1008–1016. 10.1002/bdra.20735. 20878909

[2] de Graaf G, Buckley F, Skotko BG. Estimation of the number of people with Down syndrome in the United States. Genet Med. Apr 2017;19(4):439–447. 10.1038/gim.2016.127. 27608174

[3] Ptomey LT, Szabo AN, Willis EA, Changes in cognitive function after a 12-week exercise intervention in adults with Down syndrome. Disabil Health J. 2018;11(3):486–490. 10.1016/j.dhjo.2018.02.003. 29501470 PMC6005720

[4] Dairo YM, Collett J, Dawes H, Oskrochi GR. Physical activity levels in adults with intellectual disabilities: a systematic review. Prev Med Rep. Dec 2016;4:209–219. 10.1016/j.pmedr.2016.06.008. 27413684 PMC4929079

[5] Piercy KL, Troiano RP, Ballard RM, The physical activity Guidelines for Americans. JAMA. Nov 20 2018;320(19):2020–2028. 10.1001/jama.2018.14854. 30418471 PMC9582631

[6] Hassan NM, Landorf KB, Shields N, Munteanu SE. Effectiveness of interventions to increase physical activity in individuals with intellectual disabilities: a systematic review of randomised controlled trials. J Intellect Disabil Res. Feb 2019;63(2):168–191. 10.1111/jir.12562. 30407677

[7] Zago M, Duarte NAC, Grecco LAC, Condoluci C, Oliveira CS, Galli M. Gait and postural control patterns and rehabilitation in Down syndrome: a systematic review. J Phys Ther Sci. Apr 2020;32(4):303–314. 10.1589/jpts.32.303. 32273655 PMC7113426

[8] Mahy J, Shields N, Taylor N, Dodd K. Identifying facilitators and barriers to physical activity for adults with Down syndrome. J Intellect Disabil Res. 2010;54(9):795–805. 20712696 10.1111/j.1365-2788.2010.01308.x

[9] Mitchell F, Stevens G, Jahoda A, The lifestyle behaviours of young adults with intellectual disabilities as they transition from school to adulthood: a pilot and feasibility study. J Appl Res Intellect Disabil. Nov 2018;31(6):1154–1163. 10.1111/jar.12489. 29953690

[10] Luftig RL, Muthert D. Patterns of employment and independent living of adult graduates with learning disabilities and mental retardation of an inclusionary high school vocational program. Res Dev Disabil. Jul-Aug 2005;26(4):317–325. 10.1016/j.ridd.2003.08.001. 15766626

[11] Boer PH, Moss SJ. Effect of continuous aerobic vs. interval training on selected anthropometrical, physiological and functional parameters of adults with Down syndrome. J Intellect Disabil Res. Apr 2016;60(4):322–334. 10.1111/jir.12251. 26805768

[12] Shields N, Taylor NF, Dodd KJ. Effects of a community-based progressive resistance training program on muscle performance and physical function in adults with Down syndrome: a randomized controlled trial. Arch Phys Med Rehabil. Jul 2008;89(7):1215–1220. 10.1016/j.apmr.2007.11.056. 18586124

[13] Yang C, Wickert Z, Roedel S, Time spent in MVPA during exergaming with Xbox Kinect in sedentary college students. Int J Exercise Sci. 2014;7(4):4.

[14] Melville CA, Mitchell F, Stalker K, Effectiveness of a walking programme to support adults with intellectual disabilities to increase physical activity: walk well cluster-randomised controlled trial. Int J Behav Nutr Phys Act. Sep 29 2015;12:125. 10.1186/s12966-015-0290-5. 26416606 PMC4587575

[15] McGarty AM, Downs SJ, Melville CA, Harris L. A systematic review and meta-analysis of interventions to increase physical activity in children and adolescents with intellectual disabilities. J Intellect Disabil Res. Apr 2018;62(4):312–329. 10.1111/jir.12467. 29277930

[16] McDonough DJ, Pope ZC, Zeng N, Lee JE, Gao Z. Comparison of college students’ energy expenditure, physical activity, and enjoyment during exergaming and traditional exercise. J Clin Med. Nov 10 2018;7(11). 10.3390/jcm7110433. PMC6262538 30423805

[17] Sween J, Wallington SF, Sheppard V, Taylor T, Llanos AA, Adams-Campbell LL. The role of exergaming in improving physical activity: a review. J Phys Activ Health. May 2014;11(4):864–870. 10.1123/jpah.2011-0425. PMC4180490 25078529

[18] Larsen LH, Schou L, Lund HH, Langberg H. The physical effect of exergames in healthy elderly-A systematic review. Games Health J. Aug 2013;2(4):205–212. 10.1089/g4h.2013.0036. 26192224

[19] Perrot A, Maillot P, Le Foulon A, Rebillat AS. Effect of exergaming on physical fitness, functional mobility, and cognitive functioning in adults with Down syndrome. Am J Intellect Dev Disabil. Jan 1 2021;126(1):34–44. 10.1352/1944-7558-126.1.34. 33370786

[20] Staiano AE, Calvert SL. Exergames for physical education courses: Physical, social, and cognitive benefits. Child Dev Perspect. 2011;5(2):93–98. 10.1111/j.1750-8606.2011.00162.x. 22563349 PMC3339488

[21] Lancioni GE, Singh NN, O’Reilly M, Sigafoos J, Alberti G, Desideri L. Programs using stimulation-regulating technologies to promote physical activity in people with intellectual and multiple disabilities: scoping review. JMIR Rehabil Assist Technol. Apr 7 2022;9(2), e35217. 10.2196/35217. 35389365 PMC9031065

[22] Alba-Rueda A, Moral-Munoz JA, De Miguel-Rubio A, Lucena-Anton D. Exergaming for physical therapy in patients with Down syndrome: a systematic review and meta-analysis of randomized-controlled trials. Games Health J. Apr 2022;11(2):67–78. 10.1089/g4h.2021.0172. 35438549

[23] Yang Y, Conners FA, Merrill EC. Visuo-spatial ability in individuals with Down syndrome: is it really a strength? Res Dev Disabil. Jul 2014;35(7):1473–1500. 10.1016/j.ridd.2014.04.002. 24755229 PMC4041586

[24] Silverman W. Down syndrome: cognitive phenotype. Ment Retard Dev Disabil Res Rev. 2007;13(3):228–236. 10.1002/mrdd.20156. 17910084

[25] Staiano AE, Beyl RA, Guan W, Hendrick CA, Hsia DS, Newton RL Jr. Home-based exergaming among children with overweight and obesity: a randomized clinical trial. Pediatr Obes. 2018;13(11):724–733. 10.1111/ijpo.12438. 30027607 PMC6203598

[26] Bowling AB, Slavet J, Hendrick C, The adaptive GameSquad for Xbox-based physical activity and health coaching intervention for youth with neurodevelopmental and psychiatric diagnoses: Pilot Feasibility Study. JMIR Form Res. 2021;5(5), e24566. 10.2196/24566. 33988508 PMC8164124

[27] Fernhall B, McCubbin JA, Pitetti KH, Prediction of maximal heart rate in individuals with mental retardation. Med Sci Sports Exerc. Oct 2001;33(10):1655–1660. 10.1097/00005768-200110000-00007. 11581548

[28] Crouter SE, LaMunion SR, Hibbing PR, Kaplan AS, Bassett DR Jr. Accuracy of the Cosmed K5 portable calorimeter. PLoS One. 2019;14(12), e0226290. 10.1371/journal.pone.0226290. 31841537 PMC6913985

[29] Guidetti L, Meucci M, Bolletta F, Emerenziani GP, Gallotta MC, Baldari C. Validity, reliability and minimum detectable change of COSMED K5 portable gas exchange system in breath-by-breath mode. PLoS One. 2018;13(12), e0209925. 10.1371/journal.pone.0209925. 30596748 PMC6312326

[30] Perez-Suarez I, Martin-Rincon M, Gonzalez-Henriquez JJ, Accuracy and precision of the COSMED K5 portable analyser. Front Physiol. 2018;9:1764. 10.3389/fphys.2018.01764. 30622475 PMC6308190

[31] R Core Team. R: A Language and Environment for Statistical Computing. Vienna, Austria. https://www.R-project.org.

[32] American College of Sports Medicine. ACSM’s Guidelines of Exercise Testing and Prescription. eleventh ed. Wolters Kluwer; 2022:148 (table 5.2).

[33] Pate RR, Pratt M, Blair SN, Physical activity and public health. A recommendation from the centers for disease control and prevention and the American college of Sports medicine. JAMA. Feb 1 1995;273(5):402–407. 10.1001/jama.273.5.402. 7823386

